# Volunteering *via* Smart-Phone for People With Psychosis—Protocol of a Feasibility Trial

**DOI:** 10.3389/fpsyt.2021.742202

**Published:** 2021-11-30

**Authors:** Mariana Pinto da Costa, Phone Pal Advisory Groups

**Affiliations:** ^1^Unit for Social and Community Psychiatry, Queen Mary University of London, London, United Kingdom; ^2^Institute of Psychiatry, Psychology & Neuroscience, King's College London, London, United Kingdom; ^3^Institute of Biomedical Sciences Abel Salazar, University of Porto, Porto, Portugal

**Keywords:** volunteering, smart-phones, remote, communication, digital mental health, psychosis, intervention, trial

## Abstract

The literature suggests that volunteering can be used to address social isolation and support patients with psychosis in the community. However, many expect in person meetings, requiring a greater effort of availability and commitment. There is therefore a need for more flexible, easily accessible support. Volunteering *via* smart-phone could be a useful intervention for people with psychosis. One patient and one volunteer have been matched for a duration of 12 weeks, and participants have been encouraged to communicate through a variety of communication methods (audio calls, video calls, text messages, WhatsApp messages and e-mails). The Phone Pal study aimed to investigate the feasibility of recruitment, participant retention, data collection procedures, intervention usage of the methods of communication and changes in outcome data. At baseline and follow-up outcome measures collected from patients and volunteers included their quality of life, physical activity, self-esteem and social comparison. Additional outcomes assessed patients' attachment, social contacts and symptoms; for volunteers, their social distance was evaluated. At follow-up both patients and volunteers rank their perception of their relationship with each other. This mixed method feasibility study has been conducted in two phases, the first stage evaluating a smaller sample of patients and volunteers recruited in London, and then a second phase with a larger sample of volunteers recruited from across the United Kingdom.

Trial registration: ISRCTN17586238.

## Introduction

Health-promotion interventions that increase engagement in lifestyle changes can offer potential health benefits across the lifespan, including to people with severe mental illness (SMI) (1). Amongst such interventions, one-to-one face-to-face volunteering in mental health, which already exists in the community, can be a way to promote social relationships in patients and positive attitudes toward people with SMI in volunteers (2, 3). Current evidence indicates improvements in patients' and volunteers' physical and mental health (4, 5). In spite of this, existing volunteering programmes seem to be inflexible (6) and disregard people's preferences and the challenges they encounter to physically meet (e.g., long distances and busy agendas); how modern technology is integrated into everyday life is overlooked.

A study investigating the choice of technology-based communication tools (e.g., e-mail, phone or face-to-face modalities used by the general population to communicate with their closest ties) described it as dependent on their skills, modality availability and location of their contacts (7). Patients with psychosis increasingly own technological devices such as mobile phones (8) and are using them to digitally connect (9). A meta-analysis of people with symptoms of psychosis revealed that the prevalence of phone ownership was rapidly increasing, with 81.4% ownership amongst those surveyed in 2014 and 2015 (10). Digital tools may enhance how patients and volunteers establish and maintain a relationship in a volunteering programme. This may have a positive effect in the level of community involvement, connecting people with SMI with others, such as volunteers. For people with psychosis, who often fear and avoid social interaction, speaking to a volunteer they do not know over the phone and engaging in mutual support toward a healthy lifestyle may encourage them to find new ways to interact, be reciprocal, establish more secure attachments and become more physically active. Volunteering provided remotely over smart-phones could be a route to these improvements.

To explore this, the “Phone Pal” intervention has been designed following the person-based approach and the Medical Research Council (MRC) framework for the development and evaluation of complex interventions (11). The “Phone Pal” enables patients to use a smart-phone to communicate with a volunteer through text, WhatsApp messages, e-mails, audio or video calls, thus enabling the participant to conduct informal conversations and promoting mutual encouragement toward a healthy lifestyle for a duration of up to 12 weeks (12).

According to the MRC guidelines, a feasibility study should be the next step to elucidate how the intervention works in practice. It is typical for feasibility studies to use more flexible methodology, such as an observational design, since the aims focus on evaluating acceptability and feasibility of intervention and study procedures (13). It is therefore unnecessary to use control groups and to randomize participants at this early stage in the intervention development process, although this may be necessary in a later pilot study (13) that precedes the randomized controlled trial (RCT). It is therefore important to establish the perceived acceptability of this intervention, and to evaluate the feasibility of conducting a trial.

### Aim and Objectives

The aim of this mixed methods feasibility study has been to address the uncertainties regarding the feasibility of the intervention and the study procedures.

The specific objectives have been as follows:

- To evaluate the feasibility of the following study procedures:
  – recruitment, including time to recruit
  – eligibility criteria and resulting sample characteristics
  – matching patients and volunteers
  – study retention and follow-up
  – data collection procedures and outcome measures, including missing data.
- To explore the usage of the intervention in terms of:
  – acceptability of, and adherence to, the intervention
  – patterns of intervention use.
- To investigate the acceptability of, and participants' response to the intervention by assessment of:
  – changes in outcome data and estimate of the variability of outcome measurement
  – participants' views and experiences of the intervention relating to the initial training received, access to support throughout the study, and the wider study procedures.

## Methods

### Design

A single center, pre-post, single arm, mixed methods feasibility study with two phases. The first with a small sample of patients and volunteers recruited in London; the second phase incorporating a larger sample with volunteers recruited nationwide.

A pre-post design with some process measures was chosen as the most appropriate method for addressing the study objectives. Since this has been within the initial stage of intervention development and testing, a single center was deemed as the most practicable setting. The follow-up involved quantitative assessments, data usage analysis and in-depth qualitative interviews. This evaluation planned to cover the perspective of the participants (i.e., patients and volunteers), and a system analysis of smart-phone data usage collected by two apps “mspy” and “accupedo pedometer—step counter”. The utilization of multiple methods allows for a more complete and thorough understanding of feasibility questions in the target population (14, 15), using a combination of self-reported and observed behavioral measures (16).

The first phase included 6 participants (*n* = 3 patients, *n* = 3 volunteers). If feasible, and allowing for further refinements provided by participants' feedback, a subsequent full study with another 30 participants (*n* = 15 patients, *n* = 15 volunteers) would be carried out.

### Study Materials

The study title “Phone Pal” was incorporated throughout all materials relating to the study. The researcher's goal was to use attractive and relevant imagery for the logo and study materials, which the lead author designed personally and with the objective of approximating the principle of similarity (17). The patients' and volunteers' advisory groups provided feedback to the study advert design, i.e., to make them interesting and appealing, and advised on the wording of the participant facing documents to ensure that the information was clear and understandable.

### Ethical Approval

The regulatory approvals to conduct this study were required by the lead author in her role as Chief Investigator (CI) and local Principal Investigator (PI), with the Health Research Authority (HRA) and local governance office of East London NHS Foundation Trust (ELFT).

The lead author presented the study to the East of England—Cambridgeshire and Hertfordshire Research Ethics Committee (REC reference: 18/EE/0196) on 12 June 2018. HRA approval was received on 7 September 2018. The study was then registered in the International Standard RCT Number (ISRCTN) database (ISRCTN17586238).

Further to the first stage of this study, and given the wide expression of interests received from volunteers from outside London, a minor amendment was requested on 20 February 2019 and approved on 1 March 2019 to recruit volunteers across the country, and to have support from other researchers to undertake participants' assessments.

### Recruitment and Sampling

**Figure 1:**
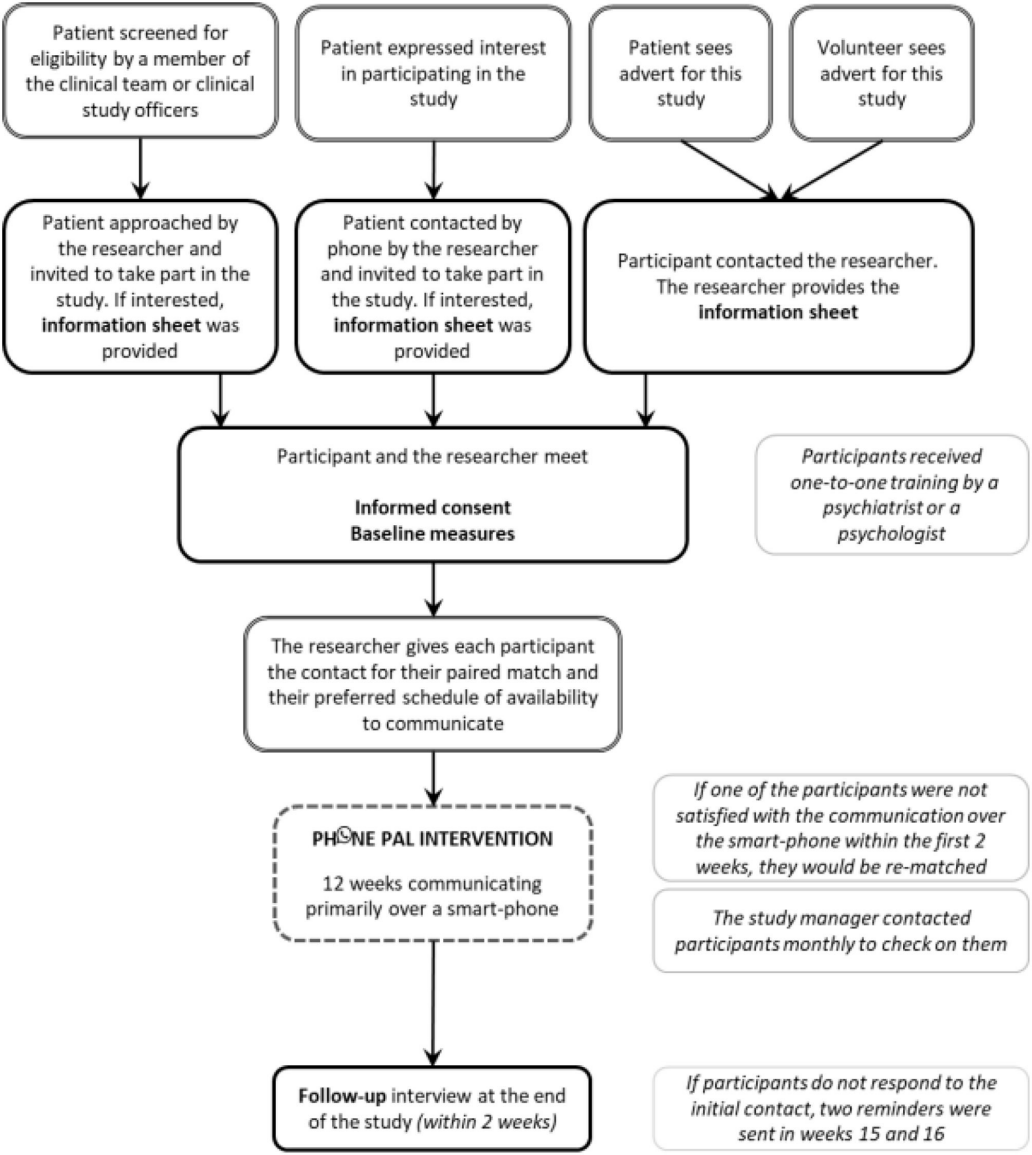
Diagram of study procedures.

Owing to the nature of the intervention, aiming to connect people in the community, it was most appropriate to recruit people with psychosis who are followed in outpatient community services, together with community volunteers. A range of recruitment strategies have been used (Figure 1). All patients have been recruited from ELFT. Volunteers have been recruited in London for the first phase of the study, and across the country for the subsequent phase.

### Study Set-Up

#### General Procedures

Preparations were made well in advance, recruitment activities were discussed with the NHS Trust and the volunteering associations at the development stage of this work.

#### Researchers' Training

Scripts were developed containing key instructions for approaching potential patient participants, and for training them on the intervention usage. The lead author provided these to the two researchers trained by the lead author to provide support with patient assessments.

In addition, all researchers received training in the Brief Psychiatric Rating Scale (BPRS) in order to be able to assess patients. The training consisted of: (i) formal teaching with the support of PowerPoint presentations that introduced the BPRS rationale, interview characteristics, description and scoring items, and (ii) video-training comprising four video-taped BPRS clinical case interviews with reference “standard” ratings previously defined. For quality assurance each BPRS interview was discussed with the lead author.

#### Patient Participants

The study was set up in ELFT, with the lead author as local Principal Investigator (PI). The lead author attained approvals through NOCLOR research support (www.noclor.nhs.uk) from three separate local boroughs, i.e., City and Hackney, Tower Hamlets and Newham to recruit people with psychosis followed in these community mental health teams (CMHTs).

Study adverts were given in person or sent by e-mail to clinicians and clinical study officers and were also placed in the waiting areas of clinical settings in CMHTs in ELFT.

#### Volunteer Participants

The study was disseminated through adverts, mailing lists and social media. Study adverts were placed in East London through flyers, posters displayed in public spaces in community venues, e.g., local libraries, charities, community centers and universities. The study was also advertised by e-mail *via* mailing lists or e-newsletters of volunteering organizations or universities or distributed online *via* social media including Twitter and Facebook.

In the first and second phases of this study, volunteers were recruited in London and from across the country, respectively.

### Eligibility Criteria

#### Inclusion Criteria

##### Patient Participants

- 18 years or over
- Clinical diagnosis of schizophrenia or a related psychotic disorder (ICD 10: F20-29)
- Interested in having a volunteer with whom they would be in contact primarily through a smart-phone for 12 weeks
- Receiving care in secondary NHS mental health services
- Have the capacity to provide informed consent
- Sufficient command of English to complete the measures.

##### Volunteer Participants

- 18 years or over
- Interested in having a patient with whom they would be in contact primarily through a smart-phone for 12 weeks
- Have the capacity to provide informed consent
- Sufficient command of English to complete the measures.

#### Exclusion Criteria

##### Patient and Volunteer Participants

- Failure to meet any of the inclusion criteria
- Unable to use smart-phones even if provided with appropriate assistive technology.

### Screening and Initial Approach

A screening log has been maintained throughout the study, using Microsoft Excel 2010, to monitor participants' progress and the feasibility of recruitment and retention.

#### Patient Participants

Potential patient participants have been identified by the clinical team, the clinical study officers and the researchers or could self-refer.

- Clinicians have been provided with the study information sheet containing the contact details of the researcher and have been encouraged to approach patients with psychosis directly. Clinicians informed patients about the study during routine clinical meetings and invited patients to take part.
- Clinical study officers screened RiO, the electronic patient record system used at ELFT, for patients with psychosis. On meeting these patients in person in CMHTs, they invited them to take part in the study. Clinicians and clinical study officers could pass on patient details to the researcher or could encourage patients to self-refer.
- A purposive sample of patients from ELFT outpatient services who had already expressed willingness for future contact in future research were contacted over the phone by the lead author and invited to take part in the Phone Pal study.
- Patients that saw the advert in the CMHT could also self-refer to the study. After speaking with the patients over the phone, the researcher would check in RiO to establish whether the patient met the study inclusion criteria, and then contact patients back to confirm.

Over the phone, the researcher explained the content of the study, covering the information described in the information sheet. Patients were invited to take part and were provided with an opportunity to discuss the study and to ask questions. Patients that were interested in the study were then invited to meet the researcher in person.

#### Volunteer Participants

- Potential volunteer participants that saw the advert and were interested in the study have been able to self-refer and to contact the researcher over the phone or *via* e-mail to express their interest.

Over the phone, the researcher explained the content of the Phone Pal study, covering the information described in the information sheet. Potential volunteers were invited to participate and were provided with an opportunity to discuss the study and ask questions. Information sheets were sent *via* e-mail. Individuals that met the inclusion criteria and maintained interest in the study were invited to meet the researcher in person.

### Consent and Enrolment

#### Patient Participants

In the face-to-face interview with the researcher, the patient was taken through the study information sheet and received detailed explanations about the intervention. It was ensured that they understood what their participation involved and that they agreed that their study-provided smart-phone communication with the volunteer was going to be checked (i.e., for patterns and frequency of the audio and video calls, and the content of any written messages).

At this initial face-to-face meeting, which occurred at ELFT facilities or other location of their convenience, participants also provided written informed consent. As further described, this was followed by the collection of baseline measures including communication usage and preferences, training and receiving £10 as a token of appreciation. The patient participant was then enrolled in the study and paired with their matched volunteer.

#### Volunteer Participants

In the face-to-face interview with the researcher, each individual was taken through the study information sheet, and received detailed explanations about the intervention and their role as a volunteer. They were also assessed for their suitability to participate. It was ensured that they understood what their participation involved and that their study-provided smart-phone communication with the patient was going to be checked (i.e., for patterns and frequency of the audio and video calls, and the content in any written messages).

At this initial face-to-face meeting, which occurred in the facilities of ELFT, Queen Mary University of London (QMUL), or other location of their convenience, participants provided written informed consent. This was followed by the collection of baseline measures, including communication usage and preferences, training and receiving £10 as a token of appreciation. The volunteer participant was then enrolled in the study and paired with their matched patient.

### Training

#### Patient Participants

In the first phase of the study, the lead author provided individual one-to-one face-to-face training for all patient participants, which lasted between 1 and 2 h, responding to the individual queries of each patient participant. In the full study, two researchers supported the lead author in enrolling the patients, conducting baseline measures, providing them training and performing the follow-up measures and interviews.

Patients' training covered an overview of the study, the intervention and its aims, the role and responsibilities of the volunteer, and guidance to engage and interact with their paired volunteer. It also included communication and listening skills, suggestions for the conversation content, confidentiality and when this could be breached, relationship boundaries, and contact information for them to have access to support and help in an emergency. The printed handouts of this training presentation were given to patient participants to keep.

On completion, patients received a smart-phone, and were trained and guided in its use by sending a text message, a WhatsApp message and an e-mail to the lead author's study phone. Patient participants were provided with the details of the Gmail account that was created for them.

#### Volunteer Participants

Volunteers' training covered an overview of the study, the intervention and its aims, the role and responsibilities of the volunteer, and guidance to engage and interact with their paired patient. It also included communication and listening skills, suggestions for the conversation content, confidentiality and when this should be breached, relationship boundaries, the potential communication and behavior problems of patients with psychosis, procedures to assess risk and to safeguard vulnerable people and report abuse, and contact information to have access to support and help in an emergency. The printed handouts of this training presentation were given to volunteer participants to keep.

This training was provided individually to all volunteers by the lead author and lasted between 1 and 2 h; all individual queries were answered. At the end of the training the volunteers were asked to sign a confidentiality agreement.

On completion, volunteers received a smart-phone, and were trained and guided in its use, testing it in the same way as the patients (see above). Volunteer participants were provided with the details of the Gmail account that was created for them.

### Matching Patients and Volunteers

The lead author matched patients and volunteers in a pragmatic way, matching the first patient with the first volunteer available throughout the recruitment period. Each volunteer only had one assigned patient.

Once they were matched, participants were sent a text message in which they were given their match contact details, i.e., study phone contact number and e-mail account details. They also received information about the general availability of their paired match in a typical week, with their preferred hours to communicate.

Participants were matched on the same day as the face-to-face meeting with the researcher, or as soon as possible and within a maximum of 2 weeks. If in the first 2 weeks after being matched, one of the participants was not satisfied with the communication with their match, the lead author would re-match those participants within a further 2 weeks.

### Access to Support and Supervision

All participants were given the study contact phone number and e-mail of the lead author. Throughout the study, the researchers followed the “ELFT Policy for Safeguarding Vulnerable Adults” and the department's “Lone Workers Policy”.

The lead author proactively contacted all participants once per month to check on them. In addition, during the course of the study, participants had access to support and supervision from the lead author for any questions or concerns; they could also book a face-to-face appointment with the lead author if required on weekdays during working hours. This could be useful if during the communication with their match, participants had heard or read something that they found emotionally distressing. Participants were informed about the mental health crisis and emergency contacts that they could call in case they had a mental health emergency, when they could be placing themselves or others at risk.

### Follow-Up

At the end of the 12 weeks, participants were telephoned to arrange a follow-up interview and asked to meet the researcher within a 2 week interval. If participants did not respond to the phone call, they were sent an SMS. In order to acknowledge participants' time, £10 was provided for completion of each assessment, i.e., at baseline and follow-up in alignment with research suggesting that this level of compensation can increase follow-up rates in digital trials (18).

### Withdrawals

Participants could decide to drop out of the study at any time. Participants who wanted to withdraw from the intervention would be asked if they also wanted to withdraw from follow-up data collection. Those that refused to take part in further data collection would be withdrawn, but their existing data would be included in the analysis unless they requested otherwise. Reasons for withdrawal would be documented.

### Sample Size

A formal sample size calculation was not conducted since the guidance on sample sizes for feasibility studies is diverse (19), although commonly adopting a figure of 30 (20). The lead author aimed to recruit 6 participants (*n* = 3 patients and *n* = 3 volunteers) in the first phase, and an additional 30 participants (*n* = 15 patients and *n* = 15 volunteers) in the second phase. These numbers were based on a pragmatic assessment; a sample of 36 participants was deemed still manageable for the lead author, whilst allowing for estimates of variability in outcome measurements and trial parameters. Of note is that this feasibility study was not designed for testing effectiveness of the intervention with a pre-determined effect size (20, 21).

### Intervention

The intervention has been described in detail elsewhere (11). Briefly, the intervention consists of a patient-volunteer pair conducting informal conversation with each other over a smart-phone through SMS, WhatsApp messages, e-mails, audio or video calls. The intervention was delivered as being flexible and low-intensity, whereby each patient-volunteer pair could determine the extent to which they would communicate, and through which smart-phone communication methods. Participants were encouraged to make contact at least once a week for a period of 12 weeks.

### Measures

Table 1 outlines the measures used in the Phone Pal study. All the questions and measures queried at baseline and follow-up are contained in the patients' and volunteers' Case Report Forms (CRFs).

**Table 1:** Measures used at baseline and follow-up.

**Assessment**	**Baseline (T1 = week 0)**	**Follow-up (T2 = week 12)**
**All participants**		
Socio-demographics	✓	
Smart-phone preferences usage	✓	✓
Availability	✓	
Quality of life (MANSA)	✓	✓
International Physical Activity Questionnaire (IPAQ)	✓	✓
Self Esteem Rating Scale—short form	✓	✓
Social Comparison Scale	✓	✓
Interviews		✓
**Patients**		
Revised Adult Attachment Scale (RAAS)	✓	✓
7-days Social Contacts Assessment	✓	✓
Brief Psychiatric Rating Scale (BPRS)	✓	✓
Scale to Assess Therapeutic Relationships—Patient Version (STAR-P)		✓
**Volunteers**		
Social Distance Questionnaire	✓	✓
Scale to Assess Therapeutic Relationships—Volunteer Version (STAR-V)		✓

The following scales were used in both patients and volunteers:

- *Subjective quality of life* has been assessed by the Manchester Short Assessment of Quality of Life (MANSA) (22), which is a 16-item scale. The instrument assesses satisfaction with life as a whole and additionally in 11 specific domains, i.e., employment, financial situation, friendships, leisure activities, accommodation, personal safety, people living in household/living alone, sex life, relationship with family, physical and mental health. Satisfaction is rated on a 7-point rating scale where 1 = could not be worse and 7 = could not be better. The mean of the 12 subjective satisfaction items is taken as the subjective quality of life score; total values can range from 12 to 84. The higher the score the better the quality of life. In addition, MANSA has 4 yes/no questions related to objective aspects of social life, i.e., having a close friend or seen a friend or safety, i.e., been accused of a crime or a victim of physical violence. The satisfaction ratings of the scale have adequate reliability with a Cronbach's α of 0.74.
- *Physical activity* has been measured with the International Physical Activity Questionnaire (IPAQ) Short-Form (23). This is a 7-item scale that assesses the types of physical activity, i.e., vigorous activities, such as aerobics; moderate activities, such as leisure cycling; walking and sitting over the last 7 days. The values of the physical activity have a maximum range of 960 min (16 h) where higher values should be excluded from the analysis, and a minimum range of 10 min where lower values should be recoded to zero. The total weekly physical activity is estimated by weighting time spent in each activity intensity with its estimated metabolic equivalent (MET) energy expenditure (24) yielding ‘metabolic equivalent minutes' per week (MET minutes/week). The IPAQ scoring protocol assigns the following MET values to walking, moderate, and vigorous intensity activity: 3.3 METs, 4.0 METs, and 8.0 METs, respectively, to report it as a continuous variable (24) (www.ipaq.ki.se). Reliability and validity, calculated in a sample of outpatients with the clinical diagnosis of schizophrenia, reported correlation coefficients of 0.68 and 0.37 for criterion validity of the reported minutes of physical activity (25).
- *Self-esteem* has been measured with the Self-Esteem Rating Scale Short-Form (SERS-SF) (26), which was adapted from the Self-Esteem Rating Scale (27) and covers different aspects such as competence, perceived self-worth and social relations. This scale consists of 20 items rated on a 7-point rating scale (1 = never to 7 = always), of which 10 items are scored positively and 20 negatively. The scale offers both positive and negative scores as well as global self-esteem scores. Positive scores correspond to a more positive self-esteem, and negative scores are indicative of more negative levels of self-esteem. SERS-SF has an internal consistency alpha coefficient of 0.91 for positive self-esteem and 0.87 for the negative scale (26).
- *Self-perceptions of social rank and relative social standing* has been assessed through the Social Comparison Scale (28). This is an 11-item scale where participants are required to make a global comparison of themselves in relation to other people and to rate themselves along a 10-point scale. Scores are obtained as a sum of all items and range from 11 to 110. Low scores point to feelings of inferiority and low rank self-perceptions. The scale has been found to have good reliability, with Cronbach alphas of 0.88 and 0.96 with clinical populations and 0.91 and 0.90 with student populations (28).

In addition, the following scales have been used for patients:

- *Close interpersonal relationships* have been assessed through the Revised Adult Attachment Scale—Close Relationships Version (RAAS) (29). This is an 18-item scale ranked on 5 points where 1 = not at all characteristic of me, and 5 = very characteristic of me. This scale measures three subscales: closeness, dependency and anxiety, each one with six items. Close refers to comfort with intimacy and emotional closeness, e.g., “I find it relatively easy to get close to people”. Depend reflects the extent to which one trusts and relies on others, e.g., “I am comfortable depending on others.” Anxiety relates to fears of rejection and abandonment, e.g., “I often worry that other people don't really love me.” A secure person should score high on the close and low on the dependency and anxiety dimensions (30). The internal consistency of the subscales has been proven using both non-clinical and clinical samples with Cronbach's α of 0.81 and 0.84 for closeness, 0.78 and 0.76 for dependency, and 0.85 and 0.90 for anxiety, respectively (29, 31).
- *Social contacts* in the past week have been assessed through the 7 days Social Contacts Assessment (32). This scale measures the number of social contacts in the past week, face-to-face or remotely, e.g., audio call, video call, e-mail, text messages or social networking, excluding people with whom participants live or mental health professionals or work contacts. The overall score reported is the number of social contacts that each patient had in the last 7 days. The psychometric properties of this scale have not been examined, although it has been widely used in other research studies with patients with psychosis (6, 33, 34).
- *Symptomatology* has been assessed through the Brief Psychiatric Rating Scale (BPRS) utilizing the 24-item version (35). The existence and severity of each of the 24 symptoms were rated on a scale where 1 = not present and 7 = extremely severe. The sum score of all 24 items reflects the symptom level, with total scores ranging from 24 to 168 and where higher scores reflect more severe psychopathology. In this study a four component analysis was chosen with four subscales: (i) depression (with the items: anxiety, depression, suicidality, guilt), (ii) manic symptoms (with the items: motor hyperactivity, elevated mood, excitement, distractibility and grandiosity), (iii) negative symptoms (blunted affect, motor retardation, emotional withdrawal and self-neglect), and (iv) positive symptoms (bizarre behavior, unusual though content, disorientation, hallucinations and suspiciousness) (36). Psychometric investigations of different BPRS versions provided evidence for satisfactory to excellent inter-rater reliability (35). There is also evidence for satisfactory validity based on score correlations with other rating scales (37) and longitudinal sensitivity to changes in psychiatric symptoms (38).
- *Character of the relationship with the volunteer* through the Scale to Assess Therapeutic Relationship—Patients Version (STAR-P). This was an adaptation of the STAR scale (39) which was developed to assess the clinician-patient therapeutic relationship in community mental health care and has patient (STAR-P) and clinician versions (STAR-C). In this study, the same items are used, but applied to characterize the relationship between patient and volunteer in order to capture relevant concepts of this relationship, e.g., trust, respect, openness and commitment. This is a 12-item scale with 5 Likert items, i.e., 1 = Never, 5 = Always. In STAR-P there are three subscale scores of positive collaboration, positive volunteer input and non-supportive volunteer input, (items that should be reversed). The total score is obtained by the sum of all items and ranges from 0 to 48, with higher scores indicating a stronger relationship between each pair. The test–retest reliability for the original scale of STAR-P was r = 0.76 with an acceptable internal consistency, i.e., Cronbach's α > 0.65 (39).

For volunteers, the following scales have been used:

- *Attitudes toward people with mental illness* through the Social Distance Questionnaire (40), a modified version of the Bogardus Social Distance Questionnaire (41). This assesses 7 areas, i.e., renting a room, being a worker, a neighbor, caretaker of the children, marrying their children, introducing to a young woman they are friendly with, recommending for a job working for a friend. It uses a 4-item Likert scale, i.e., 0 = definitely willing, 1 = probably willing, 2 = probably unwilling, 3 = definitely unwilling. The total score is obtained by the sum of all items. Higher scores represent greater desire to distance oneself from people with mental illness. The internal consistency reliability Cronbach's α of this measure was 0.92 (40).
- *Character of the relationship with the patient* through the Scale to Assess Therapeutic Relationship—Volunteer Version. This was an adaptation of the STAR scale (39) from the clinician version (STAR—C). This is a 12-item scale with 5 Likert items (1 = Never, 5 = Always). In the Volunteers version (STAR-V) there are three subscale scores: positive collaboration, emotional difficulties (items that should be reversed) and positive volunteer input. The total score is obtained by the sum of all items and ranges from 0 to 48, with higher scores indicating a stronger relationship between each pair. Test–retest reliability was *r* = 0.68 for the clinician version STAR-C, with an acceptable internal consistency, i.e., Cronbach's α > 0.65 (39).

#### Socio-Demographics

At baseline, patients were asked about their socio-demographic data and clinical information. Patients were questioned about their age, gender, marital status, country of birth, nationality, first language, ethnic background, years of education, highest level of education achieved, employment status, occupation, monthly income, who they lived with, type of accommodation, if they had children and if they had any religious or spiritual beliefs. Clinical information concerning their psychiatric treatment, i.e., clinical diagnosis, number of years with the diagnosis, hospitalisations in the past year, was also collected.

At baseline, volunteers were asked about their socio-demographics, previous experience in volunteering as well as whether they had lived experience of mental illness. The same personal socio-demographic data was then collected as for the patients. Volunteers were also questioned about their previous experience of volunteering, and if present, whether it was in mental health. In addition, volunteers were asked if they had mental health lived experience, and if so, if they had ever received any mental health treatment and hospitalisations.

#### Smart-Phone Preferences Usage

At baseline, patients and volunteers were questioned about their former smart-phone usage, and whether they had used or owned a smart-phone before. They were asked about which communication methods they most frequently used or would like to use, e.g., audio calls, video calls, text, Facebook or WhatsApp messages, e-mails or others.

At follow-up, both patients and volunteers were questioned about the communication methods they had used the study smart-phone most often for, i.e., audio calls, video calls, text messages, WhatsApp messages or e-mails, and in particular, utilized to communicate with their match.

#### Availability

In the baseline assessment, patients and volunteers were questioned about their usual availability to get into smart-phone contact with their match according to a weekly schedule.

#### Smart-Phone Usage Data

The smart-phones provided had two apps installed: “mspy”, to monitor communication and “accupedo” to monitor step count. The former aimed to collect the date and time of participants' communications through the smart-phone, to look at written message content and the frequency and duration of the audio communication. The latter aimed to collect the number of steps as recorded *via* the smart-phone pedometer app.

A database in Microsoft Excel in 2010 was used to organize participants' details of the communications retrieved and the step count number.

### Risk Assessment and Adverse Events

The patients' assessment included a health outcome symptom rating scale (BPRS) measured at baseline and follow-up. If some of the clinical features were present that would be classified as risk, e.g., suicidality, this would be addressed accordingly in line with ELFT clinical safeguarding and incident procedures. If patients endorsed this item or if the researchers were made aware of any risk during the initial assessment, patients would be advised to contact a health professional immediately and would be followed-up with a telephone call, which is in keeping with recommendations for when suicidal ideation is expressed (42).

If an adverse event (AE) would arise during smart-phone communication, it was the participant's responsibility to contact the lead author. She would then follow up the AE with the participant, establishing whether the AE had been resolved or continued, and record the event in the AE log. When the AE occurred or was identified during the assessment, then it was the researcher's responsibility to follow the same procedure. The AE would be assessed to establish whether or not it should be classified as a serious adverse event (SAE).

A SAE would be classified as: (i) “related”, when it resulted from the administration of any of the research procedures, and (ii) “unexpected”, when the type of event is not listed in the protocol as an expected occurrence. A SAE that is considered to be related and unexpected would be reported to the sponsor within 24 h of learning of the event and to the main REC within 15 days in line with the required timeframe.

### Data Analysis

The quantitative data would be analyzed through descriptive analysis for participants who completed the baseline and follow-up measures, regardless of whether they completed the intervention or withdrew (“intention to treat” analysis). Outcome measures would be assessed for completeness and the percentage of missing responses reported. To enable the calculation of the overall scales, individual mean imputation would be performed, imputing the calculated mean for a participant to the responses to the other questions (43).

Normally, in a single-group feasibility study, only a within-group estimate is possible (44), reporting the mean and standard deviation. Owing to the design of the study and the small number of participants, it is not appropriate to test for differences of effect of the intervention in the different measures. The secondary outcomes should be reported with the participants' mean scores and standard deviations in the different time points, i.e., baseline and follow-up or the median and the interquartile ranges, where appropriate.

The qualitative data from the semi-structured interviews would be analyzed through thematic analysis (45).

## Discussion

This is the first study to investigate volunteering *via* smart-phone for people with psychosis, exploring the feasibility of recruitment, retention and data collection procedures, the usage of the intervention, and the acceptability of, and participants response to the intervention.

Wide inclusion criteria were employed in line with one of the study objectives, which was to assess how acceptable the intervention was. No pre-defined stop-go criteria were set, since the purpose has been to identify any potential barriers and facilitators to performing a larger trial relating to this intervention. Commonly, progression criteria can range and encompass figures of recruitment, retention, programme implementation, achieved measures, fidelity, factors affecting protocol adherence and acceptability (46).

Importantly, this study uses only two discrete temporal assessments, at the beginning and end of the study. Only one follow-up limits understanding of how participants' outcomes may change with time and whether those changes are sustained. Future research should consider additional time points for follow-ups.

Other studies reported unplanned absences from volunteers and high levels of volunteer attrition (47, 48). It has been suggested that volunteers may drop out when there are discrepancies between “ought” and “actual experiences”. When these discrepancies between expectations and reality arise, feelings of anger and disappointment may set in, and to preserve their positive self-feeling, devoted volunteers may drop out (49). The importance of the self-regulation between volunteers and the organization in the decision to drop out or persevere has been previously recognized (49).

The lead author aimed to develop a team mind-set of the “Phone Pal study” with the two researchers that have been helping in the study. A team-based approach is important in establishing a cohesive longitudinal research framework (50, 51). Some have argued that successful follow-up is both top-down and bottom-up driven, requiring efforts from all staff, including the PI (52), which has a key role in modeling professional communication and perseverance (53), and is commonly in a position to empower staff to improve team effectiveness (54).

It has been raised that during intervention development, new measures may need to be designed that align with the theoretical perspectives and hypothesized mechanisms of change reflected in the intervention. If researchers move too soon to adopt an outcome measure in an RCT and the trial is not effective, the main problem may be the selection of an outcome measure that is insensitive to change or incongruent with the logic model of the intervention. Performance of feasibility studies to assess measures prior to larger trials is recommended to improve subsequent RCT data interpretation (55).

In the Phone Pal study, a range of observer rated (i.e., BPRS and self-reported outcomes) have been utilized, some of them concerning behavioral outcomes (e.g., social contacts and physical activity).

The publication of the quantitative and qualitative data of the Phone Pal study will shed light on volunteering *via* smart-phone for people with psychosis as an intervention, and whether a future trial should be conducted to explore its effectiveness and cost-effectiveness.

## Data Availability Statement

The original contributions presented in the study are included in the article/supplementary material, further inquiries can be directed to the corresponding author/s.

## Ethics Statement

This study was reviewed and approved by the Research Ethics Committee East of England—Cambridgeshire and Hertfordshire (REC reference: 18/EE/0196) and the Health Research Authority in the UK. The patients/participants provided their written informed consent to participate in this study.

## Author Contributions

MPC wrote the protocol, sought ethical approval of the study and has been the Chief Investigator and the Principal Investigator of the Phone Pal study. The Phone Pal Advisory Groups provided advice to the Chief Investigator. The authors contributed to the article and approved the submitted version.

## Conflict of Interest

The authors declare that the research was conducted in the absence of any commercial or financial relationships that could be construed as a potential conflict of interest.

## Publisher's Note

All claims expressed in this article are solely those of the authors and do not necessarily represent those of their affiliated organizations, or those of the publisher, the editors and the reviewers. Any product that may be evaluated in this article, or claim that may be made by its manufacturer, is not guaranteed or endorsed by the publisher.
